# Clinical Heterogeneity Among Preschoolers Recruited as Infants Due to Elevated Likelihood of Autism: A Sibling Study

**DOI:** 10.1111/sjop.70088

**Published:** 2026-03-09

**Authors:** Lisa L. Axelsson, Terje Falck‐Ytter, Pär Nyström, Mikaéla Andric Blom, Matilda A. Frick

**Affiliations:** ^1^ Department of Medical Sciences Uppsala University Uppsala Sweden; ^2^ Department of Psychology Uppsala University Uppsala Sweden; ^3^ Department of Women's and Children's Health, Center of Neurodevelopmental Disorders at Karolinska Institutet Karolinska Institutet Stockholm Sweden; ^4^ County of Uppsala Habilitation Services Uppsala Sweden; ^5^ Department of Psychology Stockholm University Stockholm Sweden

**Keywords:** attention‐deficit/hyperactivity disorder, autism, developmental language disorder, neurodevelopmental conditions, sibling study, sustained visual attention

## Abstract

Autism spectrum disorder (ASD), attention‐deficit/hyperactivity disorder (ADHD), and developmental language disorder (DLD) are neurodevelopmental conditions (NDCs) that share etiological factors and frequently co‐occur. Despite this, they have rarely been studied together—particularly in relation to functional outcomes. In this study, we investigate the association between the developmental pattern of sustained visual attention in infancy and number of diagnoses, and map the clinical profile of 6‐year‐old children. A cohort of 6‐year‐olds, originally recruited in infancy due to elevated (*n* = 42) or low (*n* = 7) likelihood of ASD, were assessed for sustained visual attention, diagnostic outcomes, general adaptive functioning, intellectual abilities, and language skills. Participants were grouped based on the number of NDC diagnoses (ASD, ADHD, DLD, and/or Subthreshold ASD) they received at follow‐up. We could not find statistical support for an association between sustained visual attention and number of diagnoses. Findings revealed no significant differences in adaptive functioning, intellectual abilities, or language skills between children with no diagnosis (*n* = 24) and those with a single diagnosis (*n* = 15). However, children with two or more diagnoses (*n* = 10) scored significantly lower in general adaptive functioning, intellectual ability, language production, and verbal comprehension compared to those with only one or no diagnosis. The results indicate that compared to children with only one diagnosis or no diagnosis, children with two or more diagnoses scored lower on several key functional domains, emphasizing the need to prioritize children with multiple diagnoses or confirmed functional impairment in clinical settings. Moreover, the findings indicate that a single diagnosis in preschool‐aged children should not be a stand‐alone outcome measure in sibling studies, if the goal is to identify early processes that predict meaningful differences in everyday functioning.

## Keypoints

- ASD, ADHD, and DLD frequently co‐occur, but are rarely studied together. This study applied a transdiagnostic approach to examine these neurodevelopmental conditions (NDCs) simultaneously in a sample of six‐year‐old children recruited in infancy for a sibling study about autism.
- We examined if infant sustained visual attention was associated to number of NDCs, and mapped diagnostic outcomes and functional outcomes at six years.
- Statistical support could not be found for association between sustained visual attention in infancy and number of NDCs.
- Children with two or more NDCs showed significantly lower scores in adaptive functioning, general cognitive functioning, and language abilities, compared to children with only one single NDC, who in turn, had no significant difference in any of the outcome measures in comparison with the group with no NDC.
- Findings suggest that it is important to prioritize children with functional impairments in clinical practice, and that sibling studies should not rely on a single NDC as outcome measure, when studying how early markers relate to later functional outcomes.

## 1 Introduction

Autism spectrum disorder (ASD), attention‐deficit/hyperactivity disorder (ADHD), and developmental language disorder (DLD) are childhood onset, co‐occurring neurodevelopmental conditions (NDCs) with high heritability rates and shared etiological features, although estimates of heritability and etiology are less certain for DLD (Keijser et al. 2024; Nudel et al. 2024; Rommelse et al. 2010). The NDCs are associated with a range of overlapping difficulties such as language deficits, poor adaptive functioning, and academic challenges (McGregor 2020; Redmond 2016; Sikora et al. 2012; Skeat et al. 2010). These NDCs are often studied in pairs of two, such as ASD and ADHD (Canals et al. 2024), ASD and DLD (Jin et al. 2023), or ADHD and DLD (Korrel et al. 2017), but less often studied all three together, which may be important to reflect the clinical reality.

The behavioral manifestations of these NDCs emerge gradually during infancy and childhood, which motivates the study of potential markers of NDCs, both for early identification in order to be able to offer early support when needed (Childress and Stark 2018; Eckes et al. 2023; Skeat et al. 2010), and to understand the factors contributing to individuals' developmental trajectories. One marker that has potential to explain broader transdiagnostic phenotypes is sustained visual attention, which is associated with later development of cognition, language, and socio‐communication (Kannass and Oakes 2008; Viktorsson et al. 2024; Yu et al. 2019). The high co‐occurrence and shared genetic underpinnings suggest that studies of infant siblings of ASD provide valuable insights into the early development of NDCs (Miller et al. 2019; Rommelse et al. 2010). In the present study, we used longitudinal data from infant siblings to children with ASD and explored if the number of diagnoses at 6 years was associated with the developmental pattern of sustained visual attention in infancy and toddlerhood. Additionally, we explored the clinical profile and functional outcome at the age of 6 years.

### 1.1 Sustained Visual Attention as an Early Potential Marker of Later Difficulties in NDCs

Sustained attention is highly relevant for development of self‐regulation and executive functions later in life (Brandes‐Aitken et al. 2019; Frick et al. 2018), and in the current study, we explore sustained visual attention as a potential marker of later NDCs. Many aspects of attention have been investigated in relation to ASD, such as joint attention (Bottema‐Beutel 2016; Bruinsma et al. 2004; Nyström et al. 2019), disengagement of attention (Siqueiros‐Sanchez et al. 2025; Zwaigenbaum et al. 2005), and attention towards non‐social stimuli (Hinz et al. 2024). However, fewer studies have investigated sustained visual attention as a potential marker for later difficulties common in ASD or ADHD, although there are some examples (e.g., Viktorsson et al. 2024; Vivanti et al. 2017). Approximately, the time between nine and 18 months is an important developmental period for visual attention (Ruff and Rothbart 2001). A notable example is Miller et al. (2018), who aimed to identify early predictors of ADHD and followed infants with elevated or low likelihood (LL) of ASD longitudinally. Their findings suggested that school‐aged children with clinical levels of ADHD symptoms showed a different longitudinal pattern regarding development of sustained visual attention in infancy compared to those who did not receive an ADHD diagnosis (Miller et al. 2018). The group with clinical levels of ADHD demonstrated a lack of growth between three and 24 months and with higher overall looking times early in infancy (Miller et al. 2018). Relatedly, Hendry et al. (2020) showed that a profile of reduced growth of attentional control in infants with elevated likelihood (EL) of ASD was associated with elevated ASD and ADHD traits and adaptive functioning difficulties at the age of 3 years. Despite these examples, there is no clear consensus as other studies have shown that parental reports of sustained attention during the first years of life were not significantly associated with symptoms of ADHD in mid childhood (Shephard et al. 2019).

Sustained visual attention is also relevant for later language abilities and other social domains. Even though joint attention and sustained attention are strongly correlated, Yu et al. (2019) found support for sustained attention, defined as visual attention to an object for more than 3 s, to be the most relevant predictor of a higher vocabulary. Longer sustained attention in infancy also seems to be associated with socio‐communicative development in toddlerhood, as measured with parent ratings (Viktorsson et al. 2024). However, the direct association between sustained visual attention and later development is not fully understood. For instance, sustained visual attention has been both positively and negatively associated with outcomes such as language abilities or cognitive development (Kannass and Oakes 2008). This is probably related to the development of visual attention itself, implying that the same visual attention task and the duration of sustained attention may reflect different attention processes depending on the developmental phase (Kannass and Oakes 2008).

In all, early development of sustained visual attention is important for later development of language, socio‐communication, self‐regulation, and cognitive abilities later in life—areas which are affected in ASD, ADHD, and DLD. Research on objectively assessed markers may bring clarity to whether infant sustained visual attention can be considered an early potential marker of later NDCs.

### 1.2 Diagnosing ASD, ADHD, and DLD

Although the three NDCs share some common etiological features, they have distinct diagnostic criteria; ASD is characterized by a different fashion of social communication and restricted and repetitive behaviors and interests, ADHD is characterized by symptoms of inattention and hyperactivity/impulsivity, and DLD involves difficulties in the comprehension and/or production of spoken, written, or other aspects of language processing (American Psychiatric Association 2013). Almost 40% of children with ASD also meet criteria for ADHD (Rong et al. 2021), and approximately 20% of children with ADHD also meet criteria for ASD (Hollingdale et al. 2020). The co‐occurrence with DLD is more inconclusive. Studies have often focused on different aspects of language and used different methodological approaches, but it is well established that there is an overlap between language difficulties and ADHD and ASD (Jin et al. 2023; Kessler and Ikuta 2023; Korrel et al. 2017; Schaeffer et al. 2023). Of the three NDCs of focus in the present study, DLD is the most common condition with a prevalence of approximately 7% (McGregor 2020; Norbury et al. 2016; Tomblin et al. 2018). Thereafter, ADHD has a global estimate of 5%, although estimates vary and seem to be increasing (Danielson et al. 2024; Faraone et al. 2021; Polanczyk et al. 2014), and lastly ASD has a global prevalence of 1%, also with increasing prevalence (Zeidan et al. 2022). Compared to other NDCs, DLD has long been neglected in research and in clinical services, even though it is nearly seven times more common than ASD (Bishop 2010; Kulkarni et al. 2022; McGregor 2020). Given the high co‐occurrence of NDCs, these findings suggest that a transdiagnostic approach in the research on NDCs may be more beneficial to better reflect the clinical reality, where individuals with NDCs vary regarding symptoms and needs of support (Astle et al. 2022).

A shift between the 4th and 5th edition of the Diagnostic and statistical manual of mental disorders (from DSM‐IV to DSM‐5 in 2013) entailed important changes in the diagnostic process of NDCs. In the DSM‐IV, the three NDCs could not be diagnosed together, but in the DSM‐5 their co‐occurrence is acknowledged. Further, DLD was moved from the category of communication disorders to neurodevelopmental disorders (American Psychiatric Association 1994, 2013). Another change for all three conditions was the addition of a specific criterion regarding impairment, which refers to that symptoms must lead to clinically significant impairment in an important area of functioning (American Psychiatric Association 1994, 2013). In summary, the DSM‐5 emphasizes the importance of mapping functional impairment and acknowledges the close association between ASD, ADHD, and DLD.

The criterion regarding impairment puts functional outcomes in everyday life in a new light. Not surprising, findings show that adaptive functioning, academic performance, and health‐related quality of life are substantially lower for children with a NDC than children with no NDC, and there seems to be an additive effect of having more than one NDC (Jin et al. 2023; McGregor 2020; Redmond 2016; Sikora et al. 2012). For instance, having both ASD and ADHD, compared to having only ASD, is associated with lower scores on measures of adaptive functioning and quality of life (Sikora et al. 2012). Many have studied cognitive profiles in people with ASD, ADHD, and/or DLD, but the results are not coherent. A study by Jin et al. (2023) showed significantly lower results on intellectual functioning for both ASD and DLD in comparison with children without any NDCs. While on the other hand, a meta‐analysis over cognitive profiles in people with ASD and ADHD respectively, rather showed that general intellectual functioning was within average for both groups (Wilson 2024). However specific cognitive profiles can be found or not for NDCs, general intellectual functioning seems to have a predicative value for academic performance (Marinopoulou et al. 2024).

Given the likelihood of developing secondary difficulties when having NDCs, early identification of NDCs is important. There can be a challenge to identify these conditions before the age of 3 or 4 years (Calder et al. 2024; Halperin and Marks 2019; Van't Hof et al. 2021; Zuckerman et al. 2017), which increases the importance of investigating early potential markers of later diagnostic outcome. Early identification of NDCs may facilitate early support and interventions to enhance the individual's independence and to prevent secondary difficulties (Childress and Stark 2018; Eckes et al. 2023; Skeat et al. 2010). Taken together, there is a need to explore early and objective transdiagnostic markers for all three NDCs, and given the infant‐ and child‐friendly properties of eye tracking, metrics of visual attention is a promising way forward. Against this background, the current study assesses sustained visual attention across the age of 10–18 months as a potential early marker of NDCs and maps the clinical profile and functional outcome in relation to number of diagnoses, having no diagnosis (ND), one diagnosis (1D), and two or more diagnoses (≥ 2D), in a sample of 6‐year‐old children.

### 1.3 Research Questions

1. Is the number of diagnoses at 6 years related to the developmental pattern of sustained visual attention in infancy and toddlerhood?
  Hypothesis: In line with previous findings (Miller et al. 2018; Zwaigenbaum et al. 2005), we expected that a flat developmental pattern of sustained visual attention across infancy and toddlerhood would be associated with NDCs.
2. What is the frequency of ASD, ADHD, and DLD diagnoses in a sample of 6‐year‐olds with EL of ASD recruited during infancy? Is the number of diagnoses related to the levels of general adaptive functioning, intellectual functioning, and language abilities?

## 2 Preregistration

The analysis plan and hypothesis were preregistered on Open Science Framework (https://osf.io/w5nk2). Some deviations have been made due to pragmatic and clinical reasons. First, the diagnosis DLD has been added due to its close association with ASD and ADHD. Second, instead of classifying the participants according to condition, the participants were classified according to number of diagnoses, as stratifying into subgroups for all combinations of conditions would inflate the statistical model complexity and undermine the statistical power at the expense of transdiagnostic explanatory value. Groups based on specific diagnostic outcome and co‐occurrences (e.g., ASD only, ADHD only, ASD + ADHD) were generally overlapping and too small (*n* ≤ 3) to carry meaningful information. The decision to group according to number of diagnoses was primarily a pragmatic decision due to small groups but it was also consistent with a transdiagnostic approach and reflects clinical reality, where different combinations of co‐occurrence are highly prevalent. For all details on changes that have been made, see Supporting Information.

## 3 Methods

### 3.1 Participants

Data were obtained from the longitudinal sibling study Early Autism Sweden (EASE; www.smasyskon.se) following infants with EL of ASD due to having an older sibling with ASD and infants with LL of ASD. Both groups were primarily from the greater Stockholm area. The EL group was recruited through advertisements, via clinic and habilitation services, and word of mouth. The LL participants were recruited from a birth register with the inclusion criterion that the participant had an older sibling with neurotypical development. Infants with pre‐term birth (< 36 weeks), confirmed or suspected medical problems (including uncorrected visual/auditory impairment), diagnosis of epilepsy, or known presence of genetic syndrome clearly related to ASD (in older sibling or infant) were excluded. Exclusion criteria were the same for the LL group as for the EL group, with an additional exclusion criterion regarding having a first‐degree relative with ASD, ADHD, or another NDC. The current study is based on the *N* = 50 participants (EL *n* = 42; 52.4% girls; LL *n* = 7; 28.6% girls) who participated in the 6‐year evaluation and includes data collected at 10, 14, and 18 months, and at 6 years. Sociodemographic data were collected via online questionnaires at 10 months, filled in by one parent (77.6% mothers). Of the responding parents, 70% had a university degree and 83.7% were born in Sweden (missing *n* = 1). One participant was excluded from the analyses due to change from LL to EL without fully reaching the inclusion criteria for the EL group. Thus, the final sample was *N* = 49.

### 3.2 Measures

#### 3.2.1 Sustained Visual Attention in Infancy and Toddlerhood

Sustained visual attention was operationalized as the duration of overall looking time to an area of interest covering all stimulus areas on the screen during a visual eye tracking task. Participants were shown point light animations of biological motion accompanied by sound on 50% of the trials (Falck‐Ytter et al. 2018). Each trial (16 in total) lasted 15 s (in total 4 min), and trials were shown in a pseudo‐random order across participants. Falck‐Ytter et al. (2018) explored preferential looking between the different conditions within the stimulus, but in the current study, the stimulus was used to extract a broader measure of sustained visual attention by calculating a mean percentage of looking time to the area of interest for all trials combined for each timepoint. The same stimulus was used at 10, 14, and 18 months. Looking time was measured with Tobii eye trackers (Tobii 1750 sampling at 50 Hz and TX300 sampling at 120 Hz, Tobii Technology, Danderyd, Sweden) and the eye tracking recording was preceded by a five‐point calibration procedure, which was repeated until calibration was found acceptable from visual inspection.

#### 3.2.2 Outcome Measures at 6 Years

A diagnostic team of licensed psychologists (including the first author) consensus scored each participant according to clinical best estimate diagnostic classification. All psychologists were trained in the research protocol and in pairs of two classified all participants regarding each of the following clinical diagnoses: ASD, ADHD, and/or DLD according to the DSM‐5 (American Psychiatric Association 2013), ASD according to the DSM‐IV (American Psychiatric Association 1994), or no diagnosis. As the EASE project was initiated shortly after the introduction of DSM‐5, both this and the previous DSM‐IV manuals were used to understand more about possible changes in the diagnostic process of ASD. It turned out that a substantial number of children received DSM‐IV diagnoses but not DSM‐5 diagnoses. These children will be referred to as *Subthreshold ASD* in this article. To assess the criterion regarding impairment, all available information about adaptive functioning, academic performance, and social functioning was considered.

#### 3.2.3 ASD Diagnosis

To diagnose ASD according to the DSM‐5 criteria, the diagnostic team had information available from the Autism Diagnostic Observation Schedule, Second Edition (Lord et al. 2012), the Autism Diagnostic Interview—Revised (Lord et al. 1994), 13 items from the DSM‐oriented subscale Pervasive Developmental Problems in the Children Behavior Checklist (Achenbach 1999) and the Teacher Report Form (Edelbrock and Achenbach 1984), Repetitive Behaviors Scale—Revised (Lam and Aman 2007), Social Responsiveness Scale, Second Edition (Constantino et al. 2003), The Childhood Routines Inventory—Revised (Evans et al. 2017), and clinical observations during the 72‐month visit.

#### 3.2.4 ADHD Diagnosis

To diagnose ADHD according to the DSM‐5 criteria, the diagnostic team had information available from parent and teacher ratings of the Strengths and Weaknesses of ADHD Symptoms and Normal Behavior Rating Scale (Swanson et al. 2012), the diagnostic parent interview Development and Well‐Being Assessment (Goodman et al. 2000), six items from the DSM‐oriented subscale Attention Deficit/Hyperactivity Problems in the parent rating Child Behavior Checklist (Achenbach 1999), and the teacher rating Teacher Report Form (Edelbrock and Achenbach 1984), and clinical observations during the 72‐months visit.

#### 3.2.5 DLD Diagnosis

To diagnose DLD according to the DSM‐5 criteria, the diagnostic team had information available from the New Reynell Developmental Language Scales (Edwards et al. 2011), subscale Verbal Comprehension from the Wechsler Preschool and Primary Scale of Intelligence—Fourth edition (Wechsler 2012), parental report from the Vineland Adaptive Behavior Scales—Second Edition (Sparrow et al. 2005) with focus on the Communication domain, the Autism Diagnostic Interview—Revised (Lord et al. 1994), and clinical observations during the 72‐month visit.

#### 3.2.6 Subthreshold ASD

To determine Subthreshold ASD, the diagnostic team had the same information as for an ASD diagnosis, and the category was applied when a child fulfilled criteria for ASD according to DSM‐IV but not DSM‐5. For instance, this category was applied when information from parents or school regarding symptoms or clinical impairment was lacking, but clear symptoms of ASD or clear signs of impairment were evident during the follow‐up visit. Subthreshold ASD includes Asperger's syndrome and Pervasive developmental disorder—not otherwise specified, and could not occur simultaneously as ASD in this sample. The decision to consider the Subthreshold ASD as a diagnostic category was to emphasize that this group did not display a neurotypical development, which would have been implied in the ND group.

#### 3.2.7 General Adaptive Functioning

General Adaptive Functioning (GAF) was measured with the parent interview Vineland Adaptive Behavior Scales—Second Edition (Sparrow et al. 2005). The interview consists of four indices: Communication, Daily Living Skills, Socialization, and Motor Skills that are combined into a general index for GAF, called Adaptive Behavior Composite score.

#### 3.2.8 Intellectual Functioning

Intellectual functioning was measured with the Wechsler Preschool and Primary Scale of Intelligence—Fourth Edition (Wechsler 2012). Full scale IQ was assessed from seven subtests (two from Verbal Comprehension Index, one from Visual Spatial Index, two from Fluid Reasoning Index, one from Working Memory Index, and one from Processing Speed Index).

#### 3.2.9 Language Abilities

Language abilities were measured with New Reynell Development Language Scale (Edwards et al. 2011), and Verbal Comprehension Index from the Wechsler Preschool and Primary Scale of Intelligence (Wechsler 2012). The New Reynell Development Language Scale is made up of two closely aligned scales: The Comprehension Scale that explores aspects of the child's understanding of language (hereon called language comprehension), and the Production Scale that examines the child's production of language (hereon called language production). Verbal Comprehension Index includes subtests Information and Similarities (hereon called verbal comprehension). Therefore, the measure of language abilities comprises language comprehension, language production, and verbal comprehension.

### 3.3 Analytic Strategy and Preliminary Results

Skewness and kurtosis were examined to assess the normal distribution (values between −1 and 1 were considered to not differ significantly from normal distribution). Eye tracking data for all three timepoints did not differ significantly from normal distribution. Participants with eye tracking data from only one timepoint were excluded in the analyses (ND excluded *n* = 2; 1D excluded *n* = 1; ≥ 2D excluded *n* = 5), resulting in a final sample of *n* = 41. There were missing eye tracking data from one timepoint in all groups (ND: 10 months missing *n* = 3, 14 months missing *n* = 4, 18 months missing *n* = 1; 1D: 10 months missing *n* = 4, 14 months missing *n* = 1, 18 months missing *n* = 1; ≥ 2D: 10 months missing *n* = 3, 18 months missing *n* = 1). Due to high levels of missing data, we decided not to impute data, as imputing such a large proportion of data may result in more bias. To examine if eye tracking data were missing completely at random (MCAR), little MCAR's test was performed, and the missing data were considered to be MCAR (*χ* ^2^ = 16.05; df = 10; *p* = 0.10). A linear mixed model was fitted to examine the effect of overall looking time across the three timepoints, accounting for the hierarchical structure of the data with overall looking time nested within the individual. The model included timepoint and number of diagnoses as fixed effects, and participant ID as random effect. Random intercept was included.

The frequency of ASD, ADHD, DLD and Subthreshold ASD were analyzed descriptively. For GAF, intellectual functioning, verbal comprehension, and language comprehension, data were normally distributed. Language production was positively skewed and one outlier was identified by converting scores to *z*‐scores (*z* ± 2 was considered an outlier). The outlier was replaced through Winsorizing and data reached conditions for normality. There were missing data in GAF (ND missing *n* = 1; 1D missing *n* = 2; ≥ 2D missing *n* = 1) and in New Reynell Development Language Scale (ND missing *n* = 1; 1D missing *n* = 3; ≥ 2D missing *n* = 1). Little MCAR's test was performed. The missing descriptive data were considered to be MCAR (*χ* ^2^ = 7.56; df = 7; *p* = 0.37). Expectation Maximization was performed to handle missing data. A MANOVA was performed to compare group differences for GAF, intellectual functioning, and language abilities. Tukey's HSD Test for multiple comparisons was used for post hoc analysis. All analyses were performed using software IBM SPSS Statistics, version 28.0.1.0 (142).

## 4 Results

### 4.1 The Association Between Patterns of Sustained Visual Attention and Number of Diagnoses at 6 Years

The association between developmental patterns of sustained visual attention in infancy and toddlerhood and number of diagnoses at 6 years was uncertain due to low statistical power (Table S1 and Figure S1). A visual inspection of Figure S1 indicated that the ND group and the 1D group had a similar pattern of sustained visual attention, which seemed to differ from ≥ 2D. In a similar fashion, we could not find statistical support for significant differences in sustained visual attention between number of diagnoses at any given timepoint (10 months: *F*(2, 28) = 0.95, *p* = 0.40); 14 months: (*F*(2, 33) = 0.63, *p* = 0.93); 18 months: (*F*(2, 35) = 0.41, *p* = 0.67).

**TABLE 1:** Frequency of diagnostic outcome and participant characteristics.

Diagnostic outcome	No diagnosis		ASD		ADHD		DLD		Subthreshold ASD	

*n*	%	*n*	%	*n*	%	*n*	%	*n*	%
Participant characteristics	24	49.0	6	12.2	13	26.5	6	12.2	12	24.5
Female	12	50.0	2	33.3	6	46.2	5	83.3	6	50.0
Male	12	50.0	4	66.6	7	53.8	1	16.7	6	50.0
Recruited as EL	18	75.0	6	100.0	13	100.0	6	100.0	11	91.7
Recruited as LL	6	25.0	0		0		0		1	8.3
Co‐occurrence										
Only one diagnosis	n/a		3		4		1		7	
+ASD	n/a		n/a		3		0		n/a	
+ADHD	n/a		3		n/a		2		2	
+DLD	n/a		0		2		n/a		1	
+High ASD symptoms	n/a		n/a		2		1		n/a	
+ADHD +High ASD symptoms	n/a		n/a		n/a		2		n/a	
+DLD +High ASD symptoms	n/a		n/a		2		n/a		n/a	
+ADHD +DLD	n/a		0		n/a		n/a		2	

Abbreviations: ASD, Autism Spectrum Disorder; ADHD, Attention Deficit/Hyperactivity Disorder; DLD, Developmental Language Disorder; N/a, not applicable; Subthreshold ASD, Asperger Syndrome and Pervasive Developmental Disorder Not Otherwise Specified (PDD‐NOS).

### 4.2 Diagnostic Frequencies and Levels of GAF, Intellectual Functioning, and Language Abilities

ADHD was the most frequent diagnosis within the sample (Table 1), a little more than a quarter (*n* = 13) reached criteria for ADHD, and almost 70% of the cases were co‐occurrent with another NDC. Thereafter, Subthreshold ASD was the second most frequent condition; almost a quarter of the sample (*n* = 12) showed clinical features of the condition. Almost 1/8 (*n* = 6) met criteria for ASD, and half of these cases were co‐occurrent with other diagnoses. Almost 1/8 (*n* = 6) met criteria for DLD, but only one of the participants met DLD criteria without any co‐occurring NDC. After clustering according to number of diagnoses, most of the participants belonged to ND (*n* = 24). The second largest group was 1D (*n* = 15), and the smallest group was ≥ 2D (*n* = 10). Co‐occurrence was present between ADHD and all other diagnostic outcomes, and the distribution of the different co‐occurrences was even (Table 2).

**TABLE 2:** Participant characteristics, means, standard deviations, and one‐way multivariate analysis of variance for outcome measures by six years of age.

Number of diagnoses	No diagnosis (ND)		1 diagnosis (1D)		≥ 2 diagnoses (≥ 2D)				

*n*	%	*n*	%	*n*	%			
Participant characteristics	24	49	15	31	10	20			
Female	12	50	6	40	6	60			
Male	12	50	9	60	4	40			
Recruited as EL	18	75.0	14	93.3	10	100.0			
Recruited as LL	6	25.0	1	6.7	0	0.0			

	*M*	SD	*M*	SD	*M*	SD	*F*(2,46)	*η* ^2^	Post hoc
Measure									
General Adaptive Functioning	99.55	8.78	94.67	9.33	85.60	10.54	7.95*	0.26	ND|1D > 2D (*p* < 0.001)
Intellectual Functioning	108.96	8.41	103.53	17.89	94.80	12.26	4.42**	0.16	ND|1D > 2D (*p* = 0.013)
Language Comprehension	99.13	15.78	100.11	14.87	90.50	27.02	0.98	0.04	—
Language Production	113.49	9.71	107.68	15.62	89.68	16.671	12.01*	0.33	ND|1D > 2D (*p* < 0.001)
Verbal Comprehension	108.37	15.96	98.20	26.56	80.70	17.63	11.42***	0.23	ND|1D > 2D (*p* = 0.002)

*Note:* Subthreshold ASD is included in 1 Diagnosis or ≥ 2 Diagnoses.

Abbreviations: EL, elevated likelihood; LL, low likelihood.

* *p* ≤ 0.001.

** *p* = 0.018.

*** *p* = 0.003.

For the MANOVA, a main effect of number of diagnoses across the outcome measures at 6 years was found, *F*(10,86) = 3.05, *p* = 0.002, Wilk's Λ = 0.56, partial *η* ^2^ = 0.27. Groups differed significantly from each other on four out of five measures. No group differences were found for language comprehension. The ≥ 2D group had significantly lower scores in GAF, intellectual functioning, and language production than the ND group and the 1D group. Additionally, the ≥ 2D group scored significantly lower in verbal comprehension than the ND group, but no statistical difference was found between the 1D group and the ≥ 2D group regarding verbal comprehension. No significant difference was found between ND and 1D on any of the outcome measures. Sensitivity analyses were performed without the LL participants and without imputed data (ND: *n* = 22; 1D: *n* = 10; ≥ 2D: *n* = 8) to verify that group differences were not only present due to the results of the LL group or with imputed data. The differences between groups were consistent without inclusion of the LL participants and without imputed data.

## 5 Discussion

ASD, ADHD, and DLD have a high co‐occurrence, but the three conditions have seldom been examined simultaneously. In this explorative study, we took a transdiagnostic approach (Astle et al. 2022) and examined the frequency of these conditions in a sample of 6‐year‐olds with EL of ASD. Specifically, we explored if the number of diagnoses was associated with differences in early sustained visual attention, general adaptive functioning, intellectual functioning, and language abilities. Approximately half of the sample reached criteria for one or more diagnoses. Co‐occurrence of NDCs was highly prevalent, and ADHD was the most frequent diagnostic outcome category followed by Subthreshold ASD, which supports previous findings that ADHD and ASD have partly overlapping etiology (Canals et al. 2024; Hollingdale et al. 2020; Rommelse et al. 2010). Given that the sample was recruited from having an older sibling with autism, it is not entirely unexpected that Subthreshold ASD occurred with high frequency. Results showed that individuals with two or more diagnoses had significantly lower scores on adaptive functioning, intellectual functioning, verbal comprehension, and language production than individuals with one or no diagnosis. Somewhat unexpectedly, no difference was found between having no diagnosis and having one single diagnosis. This indicates that there may be a non‐linear relationship between the number of diagnoses and several important functional outcome measures.

Our hypothesis, that the pattern of sustained visual attention would be associated with the number of diagnoses at 6 years, could not be supported in our findings. We had substantial missingness of eye‐tracking data, especially in the group with two or more diagnoses, which may have affected the results. The interpretation of these results is therefore inconclusive. Nonetheless, it is worth considering potential tendencies in the data given the explorative nature of the study. For the group with no diagnosis and the group with one diagnosis, the patterns look similar: a small increase in looking time from 10 to 18 months. Both groups seem to follow the same developmental pattern of sustained visual attention. For the group with two or more diagnoses, a slightly different pattern was implicated through visual inspection (a longer looking time at 10 months followed by a decrease in looking time by 18 months), in similarity with previous findings (Miller et al. 2018). A possible interpretation of the longer looking time in the group with two or more diagnoses is that this reflects the development of visual attention itself, which is consistent with previous findings by Kannass and Oakes (2008). This does not necessarily contradict our hypothesis, since the development of visual attention could still be linked to functional outcomes related to NDCs (Hendry et al. 2020; Miller et al. 2018; Viktorsson et al. 2024; Vivanti et al. 2017). However, the pattern that the group with two or more diagnoses had a different looking pattern than the group with no diagnosis and the group with only one diagnosis was not supported by the statistical model. We do not rule out that future studies refine the paradigm and find significant associations in larger samples.

We kept the DSM‐IV criteria for ASD, in addition to the DSM‐5, to capture subthreshold features of ASD, and even though these children did not reach the current criteria for ASD, they were not considered to be a neurotypically developing group. Indeed, the results indicate that having high ASD symptoms, in co‐occurrence with at least another NDC, was associated with significantly lower scores on important functional areas than having only ASD. This indicates that displaying ASD without co‐occurrence may not necessarily be associated with distinct impairment. Considering co‐occurring subthreshold features of ASD when also assessing ADHD and/or DLD may uncover functional impairments that extend beyond those reaching full diagnostic criteria for ASD alone. A transdiagnostic approach could be helpful to identify those with milder or fewer symptoms, but with need of support. Further, a transdiagnostic approach also highlights that functional impairment, such as low general adaptive behavior or poor language abilities, could serve as relevant indicators when prioritizing who should get referred for an assessment, instead of only considering core symptoms of NDCs. In summary, to begin an assessment with mapping functional impairment, assess with a transdiagnostic approach to NDCs, and to consider subthreshold features of ASD could be important to target the ones who need support the most and to customize interventions.

Only one participant received a DLD diagnosis with no co‐occurring condition. This indicates that DLD most often is diagnosed in co‐occurrence with other NDCs, in line with previous research showing the close correlation between DLD and other NDCs (Méndez‐Freije et al. 2023; Schaeffer et al. 2023). Even though a small sample, our results imply that DLD is less often identified as an independent diagnosis, at least in our sample where the participants were selectively recruited based on their EL of ASD.

Previous findings show that having two diagnoses, compared to having only one, is associated with lower scores on general adaptive functioning and quality of life (Sikora et al. 2012). Our results both support and make additional contributions to previous findings. On the one hand, we do see a significantly lower score on general adaptive functioning for the children with two or more diagnoses, as well as lower intellectual functioning and language abilities, than having only one diagnosis. On the other hand, we did not find any significant differences between the group with one diagnosis and the group with no diagnosis, although the descriptive data show a monotonic trend. Our results make meaningful contributions to the field of sibling studies about early markers of NDCs and indicate that a single diagnosis has only a limited effect on daily functioning. Therefore, we should not rely solely on having one single diagnosis as the outcome measure when studying how early markers relate to later functional outcomes. A transdiagnostic approach can help us to better understand how function plays an important role in addition to categorical symptoms. Furthermore, the results indicate that it is important to be especially observant if two or more NDCs might be present, not just specifically the co‐occurrence of ASD and ADHD, both in clinical settings and in research settings.

### 5.1 Limitations

We set out to investigate ASD, ADHD, and DLD together, and their associations with functional outcomes and sustained visual attention. Due to a small sample size and the high co‐occurrence, we made a pragmatic decision to group the participants according to number of diagnoses instead of grouping according to a specific diagnosis. This meant that we took a transdiagnostic approach to NDCs. To the best of our knowledge, it is novel to investigate functional outcomes and sustained visual attention as an early marker of NDCs in relation to number of diagnoses, which paves the way for the exploratory nature of the study. A limitation of this approach is that it provides little insight into the specific associations between the outcomes and diagnosis, and that the results might blur condition‐specific patterns. However, a transdiagnostic approach might reflect a clinical reality better, as children often have co‐occurring NDCs, rather than having one categorical diagnosis which is more common in research (Astle et al. 2022). Although our aim was to have a transdiagnostic approach in this study, with the inclusion of the Subthreshold ASD category and the choice to group according to number of diagnoses, this study still applied categorical analyses and not dimensional, which would have been more in line with a transdiagnostic approach to research (Astle et al. 2022).

Our sample is recruited due to EL of ASD and not recruited from clinics. It is not certain that our results would be the same if investigated in a clinical sample. The parents in the EASE project were highly dedicated to contribute to research and curious about their child's development, but we cannot conclude that this group of parents is representative of a clinical parent group. A limitation of our study is that we did not include a specific measure of social functioning. Although general adaptive functioning includes aspects of social functioning, it also includes problem solving, language abilities, and practical everyday life skills (Sparrow et al. 2005; Tassé et al. 2012), and we only used the composite score of all those domains. Social functioning and possible consequences thereof could be more evident in a clinical setting than in a research setting. Hence, clinical patients with only one NDC might have greater social impairment than what is visible in our sample.

The initial sample size was marginally insufficient, which has led to uncertainty in the interpretation of the results. Post hoc power analysis showed a power of 0.68 to detect a strong effect size, with the current group sizes and with nearly full datasets on all functional measures, which was lower than we wished for. Furthermore, the limited statistical power was most evident regarding the eye‐tracking data (particularly due to missing data for the ≥ 2D group), which resulted in an underpowered statistical model (Bell et al. 2010; Maas and Hox 2005). Due to these power limitations, we have opted for a conservative interpretation of the sustained visual attention results, mainly, that the null findings cannot be interpreted as non‐significant results but rather a consequence of low power. The plotted data suggested a slightly different developmental trajectory of sustained visual attention for the group with two more diagnoses. However, due to the low statistical power, we cannot assert that this represents a robust or reliable finding, and should be seen through an explorative lens. We encourage future studies to engage in more specific and more statistically powered analyses.

## 6 Conclusions

In summary, our findings show that having two or more NDCs is associated with significantly lower adaptive functioning, intellectual functioning, verbal comprehension, and language production, compared to having only one single NDC. In contrast, children with one diagnosis did not differ from children with no diagnosis in any important functional areas. This suggests a possible non‐linear relation between number of NDCs and functional outcomes, which future studies may test. Additionally, these differences were found across co‐occurrences of NDCs, drawing from a transdiagnostic perspective. Furthermore, considering subthreshold symptoms of ASD in the assessment of ADHD and DLD may uncover functional impairments that extend beyond those who reach full diagnostic criteria for ASD only. Our findings suggest that it is of utter importance to map functional areas and to assess possible co‐occurrence of NDCs, to be able to identify and prioritize the group with the greatest need of support. Moreover, our results can be of importance for sibling studies focusing on early markers and their relation to later functional outcomes in life. Although the sample size is small, which affected how the participants were grouped, our results propose that it is meaningful for future studies to explore further clustering based on the number of diagnoses and its associations with key functional areas, extending beyond traditional diagnostic categories.

## Author Contributions

**Lisa L. Axelsson:** conceptualization, formal analysis, investigation, writing – original draft, writing – review and editing. **Terje Falck‐Ytter:** conceptualization, resources, writing – review and editing, funding acquisition. **Pär Nyström:** software, formal analysis, writing – review and editing. **Mikaéla Andric Blom:** conceptualization, writing – original draft. **Matilda A. Frick:** conceptualization, methodology, writing – review and editing, supervision.

## Funding

This research was funded by the Riksbankens Jubileumsfond, the Knut and Alice Wallenberg Foundation, Stiftelsen Sunnerdahls Handikappfond, and the European Commission (H2020 project CANDY; grant 847818). The work was also supported by the Innovative Medicines Initiative 2 Joint Undertaking under grant agreement no. 777394. This Joint Undertaking receives support from the European Union's Horizon 2020 research and innovation program and EFPIA and AUTISM SPEAKS, Autistica, SFARI. Any views expressed are those of the author(s) and not necessarily those of the funders.

## Ethics Statement

The study was approved by the regional ethical review authority in Stockholm, Sweden, and was conducted in accordance with the Declaration of Helsinki. All caregivers provided informed consent to take part in the study.

## Conflicts of Interest

The authors declare no conflicts of interest.

## Supporting information


**Table S1:** Fixed‐effects estimates from the linear mixed model with overall looking time as dependent variable, and number of diagnoses and timepoint as fixed effects, with participant id as random intercept. SE denotes standard error of the mean.
**Figure S1:** Effect plot of the interaction between timepoint and number of diagnoses on looking time. Bars represent standard error.

## Data Availability

Due to the lack of participant consent for data sharing, the data cannot be publicly available.
