# Supplementary material for: Clinical Heterogeneity Among Preschoolers Recruited as Infants Due to Elevated Likelihood of Autism: A Sibling Study
Source: Scand J Psychol. 2026 Mar 9;67(4):1051–61. doi: 10.1111/sjop.70088 (PMC13352579; doi:10.1111/sjop.70088)
Supplement: Supplementary file 1 — Table S1: Fixed‐effects estimates from the linear mixed model with overall looking time as dependent variable, and number of diagnoses and timepoint as fixed effects, with participant id as random intercept. SE denotes standard error of the mean. Figure S1: Effect plot of the interaction between timepoint and number of diagnoses on looking time. Bars represent standard error. [file SJOP-67-1051-s001.docx]

**Clinical heterogeneity among preschoolers recruited as infants due to elevated likelihood of autism: a sibling study**

**Supplementary Information**

***Preregistration***

The analysis plan and hypothesis were preregistered on Open Science Framework (https://osf.io/w5nk2). However, some deviations have been made due to pragmatic reasons. First, the diagnosis developmental language disorder (DLD) has been added in the study due to its close association with autism and ADHD, also because we initially were not aware of that the DLD data was available. Second, language abilities were assessed using three measures: Comprehension Scale and Production Scale in New Reynell Development Language Scale (NRDLS), and Verbal Comprehension Index (VCI) from Wechsler Preschool and Primary Scale of Intelligence – Fourth Edition (WPPSI-IV). The addition of VCI deviated from the preregistered plan, which originally included only two language measures. This amendment was prompted by a correlation analysis between the subscales in NRDLS and VCI, conducted as a sensitivity check to assess the robustness of NRDLS, as NRDLS is less commonly used in both research and clinical settings compared to WPPSI-IV. Upon recognizing the availability of VCI, we incorporated it into the analysis rather than limiting its use to sensitivity analysis or excluding it altogether. Third, diagnostic outcome split into separate conditions entailed that the groups would have been too small since several of the participants had multiple conditions. Hence, instead of classifying the participants according to condition, the participants were classified according to number of diagnoses, both for the first and second research question.

| **Table S1**  *Fixed-effects estimates from the linear mixed model with overall looking time as dependent variable, and number of diagnoses and timepoint as fixed effects, with participant id as random intercept. SE denotes standard error of the mean.* | | | | |
| --- | --- | --- | --- | --- |
| Fixed effect | Estimate | *SE* | *t* | *p* |
| Intercept | 0.55 | 0.05 | 11.36 | < .001 |
| Timepoint |  |  |  |  |
| 10 months | -0.09 | 0.08 | 1.11 | .27 |
| 14 months | -0.01 | 0.06 | -0.10 | .92 |
| Number of diagnoses |  |  |  |  |
| No Diagnosis | 0.04 | 0.05 | 0.67 | .51 |
| 1 Diagnosis | 0.04 | 0.06 | 0.76 | .45 |
| Timepoint x Number of diagnoses |  |  |  |  |
| 10 months x No Diagnosis | -0.12 | 0.09 | -1.45 | .15 |
| 10 months x 1 Diagnosis | -0.16 | 0.09 | -1.76 | .08 |
| 14 months x No diagnosis | -0.03 | 0.07 | -0.50 | .62 |
| 14 months x 1 Diagnosis | -0.03 | 0.07 | -0.47 | .64 |

*Note.* Parameters (18 months, ≥ 2 Diagnoses, 14 months x ≥ 2 Diagnoses, 18 months x No Diagnosis, 18 months x 1 Diagnosis, 18 months x ≥ 2 Diagnoses) are set to zero because they are redundant in the model, and are not shown in the table.

**Figure S1.**

*Effect plot of the interaction between timepoint and number of diagnoses on looking time. Bars represent standard error.*


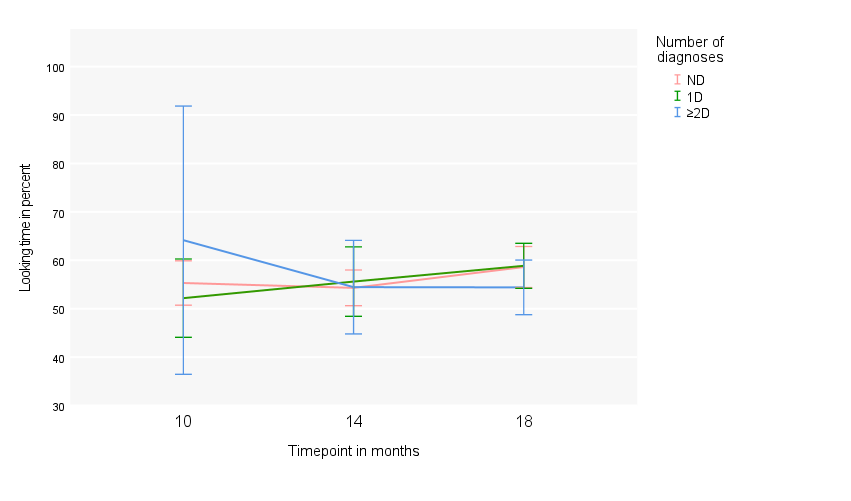


*Note.* Timepoint 10 months: ND *n* = 19; 1D *n* = 10; ≥2D *n* = 2. Timepoint 14 months: ND *n* = 18; 1D *n* = 13; ≥2D *n* = 5. Timepoint 18 months: ND *n* = 21; 1D *n* = 13; ≥2D *n* = 4.
